# A 20-Gene Signature Predicting Survival in Patients with Clear Cell Renal Cell Carcinoma Based on Basement Membrane

**DOI:** 10.1155/2023/1302278

**Published:** 2023-04-08

**Authors:** Zhenjie Yin, Yu Zhao, Weiwen Zhou, Chengcheng You, Yuanyuan Bai, Bingyong You, Dongming Lu, Shangfan Liao, Luoping Zheng, Yingming Sun, Yongyang Wu

**Affiliations:** ^1^Department of Urology, Affiliated Sanming First Hospital, Fujian Medical University, Sanming, Fujian 365001, China; ^2^Department of Medical and Radiation Oncology, Affiliated Sanming First Hospital, Fujian Medical University, Sanming, Fujian 365001, China; ^3^Hubei Key Laboratory of Tumor Microenvironment and Immunotherapy, China Three Gorges University, Yichang, Hubei 443002, China

## Abstract

**Objectives:**

The most common subtype of renal cell carcinoma, clear cell renal cell carcinoma (ccRCC), has a high heterogeneity and aggressive nature. The basement membrane (BM) is known to play a vital role in tumor metastasis. BM-related genes remain untested in ccRCC, however, in terms of their prognostic significance.

**Methods:**

BM-related genes were gleaned from the most recent cutting-edge research. The RNA-seq and clinical data of the ccRCC were obtained from TCGA and GEO databases, respectively. The multigene signature was constructed using the univariate Cox regression and the LASSO regression algorithm. Then, clinical features and prognostic signatures were combined to form a nomogram to predict individual survival probabilities. Using functional enrichment analysis and immune-correlation analysis, we investigated potential enrichment pathways and immunological characteristics associated with BM-related-gene signature.

**Results:**

In this study, we built a model of 20 BM-related genes and classified them as high-risk or low-risk, with each having its anticipated risk profile. Patients in the high-risk group showed significantly reduced OS compared with patients in the low-risk group in the TCGA cohort, as was confirmed by the testing dataset. Functional analysis showed that the BM-based model was linked to cell-substrate adhesion and tumor-related signaling pathways. Comparative analysis of immune cell infiltration degrees and immune checkpoints reveals a central role for BM-related genes in controlling the interplay between the immune interaction and the tumor microenvironment of ccRCC.

**Conclusions:**

We combined clinical characteristics known to predict the prognosis of ccRCC patients to create a gene signature associated with BM. Our findings may also be useful for forecasting how well immunotherapies would work against ccRCC. Targeting BM may be a therapeutic alternative for ccRCC, but the underlying mechanism still needs further exploration.

## 1. Introduction

Approximately 2%–3% of all adult urinary malignancies are renal cell carcinomas (RCC), which are cancers of the kidneys [1]. By 2022, It is estimated that 79,000 additional cases of RCC will be detected in the United States [2]. Clear cell RCC (ccRCC), which accounts for approximately 70%, is the most frequent subtype. Despite advancements in urology technology, the prognosis of advanced RCC remains unfavorable [3]. Therefore, exploring new biomarkers for prognosis prediction and immunotherapy for ccRCC is crucial.

The tumor microenvironment (TME), which consists of an extracellular matrix (ECM), is strongly associated with cancer development [4]. Basement membrane (BM), a widely distributed ECM, plays an important role in biological systems, such as resisting mechanical stress, dictating tissue shape, and creating diffusion barriers [5]. The main structural backbone of BM is laminin, collagen IV, nidogens, proteoglycans, and growth factors. As reported in existing studies, abnormalities in the chemical and mechanical properties of the BMs are associated with various diseases including malignant tumors [6–9]. The effect of ECM components on various RCC cell lines is heterogeneous [10], in which BM integrity can serve as a good prognostic marker in RCC [11]. Jayadev R et al. defined and created an extensive network of 224 BM-related genes and further identified their growing association with human disease [12]. Although many studies have investigated prognostic risk signatures of ccRCC previously, none have attempted to develop a prognostic risk signature with BMs in ccRCC, and it is still unclear if these BM-related genes affect patient prognosis.

In this investigation, this bioinformatics analysis was carried out in this work by creating a separate prognostic BM-related gene signature in ccRCC utilizing The Cancer Genome Atlas (TCGA) database and confirming it in the Gene Expression Omnibus (GEO) database. Then, by combining clinical data and prognostic signatures, a novel nomogram was created to predict individual survival rates. Using functional enrichment analysis and immune-correlation analysis, we investigated potential enrichment pathways and immunological characteristics linked with BM-related-gene signature.

## 2. Methods

### 2.1. Data Collection

RNA-Seq data profiles and corresponding clinical information for kidney renal clear cell carcinoma (KIRC) were downloaded from the TCGA dataset (https://portal.gdc.cancer.gov/) [13]. We also downloaded GSE29609 consisting of 39 KIRC tissues from the GEO database (https://www.ncbi.nlm.nih.gov/geo/) for validation. The RCC dataset contained 541 cancerous and 72 normal tissues, accompanied by clinical information. After that, genes associated with BM were culled from the existing literature [12] and listed in Supplementary Table S1.

### 2.2. Construction of a Prognostic BM-Related Gene Signature

To identify BM-related DEGs in the TCGA cohort in tumor and paracancer tissues, we used the limma package. To identify potentially predictive genes associated with BM, we performed a univariate Cox analysis of overall survival (OS) and displayed the results with forest plots. By performing automatic feature selection, LASSO Cox regression analysis, a method for screening signatures with generally effective prognostication performance, reduces estimated variance and avoids overfitting while providing an interpretable final model [14]. The R package glmnet was utilized for the analysis, while LASSO regression was utilized for feature selection. Using gene expression and the appropriate Cox regression coefficient, a patient's risk score was determined. Score = *e*^sum(expression of each gene × corresponding coefficient)^ was the formula developed. The patients were then classified into high- and low-risk categories based on the median risk score. To further examine the difference in OS between high- and low-risk groups, a Kaplan–Meier (KM) curve was constructed. To evaluate the predictive power of the gene signature and risk score, the time ROC (v0.4) analysis was performed.

### 2.3. Nomogram Establishment and Subgroup Analysis

The nomogram was built and calibrated using the survival and rms packages in R version 4.1.0 using the multivariable model coefficients. Harrell's concordance index (C-index) was used to evaluate the nomogram's discriminatory performance. To compile the clinical data, each participant's age, gender, race, pathological grade, T stage, N stage, M stage, and survival information were documented. We performed dichotomies based on clinical information for subgroup analysis. For continuous variables, the ROC curve is utilized to pick the appropriate cut-off value. For categorical variables, we classified them based on the AJCC stage [15], WHO/ISUP classification [16], and current research.

### 2.4. Protein-Protein Interaction (PPI) and Functional Enrichment Analyses

To learn more about the protein-protein interactions among the shared prognostic DEGs, we consulted the STRING database (http://www.string-db.org/). Moreover, gene ontology (GO) and kyoto encyclopedia of genes and genomes (KEGG) enrichment analyses were performed on the DEGs using the cluster profiler program.

### 2.5. Correlation Analysis with Immune Infiltration

Using the TIMER, CIBERSORT, XCELL, and EPIC algorithms, we explored the correlation between BM-related genes and the degree of immune infiltration. We also utilized violin plots to assess the association between the expression of high- and low-risk groups and immune checkpoints (PDCD1, CD274, CTLA-4, TIGIT, LAG3, and CD28).

## 3. Results

### 3.1. Identification of Prognostic BM-Related Genes in the TCGA Cohort

**Figure 1:**
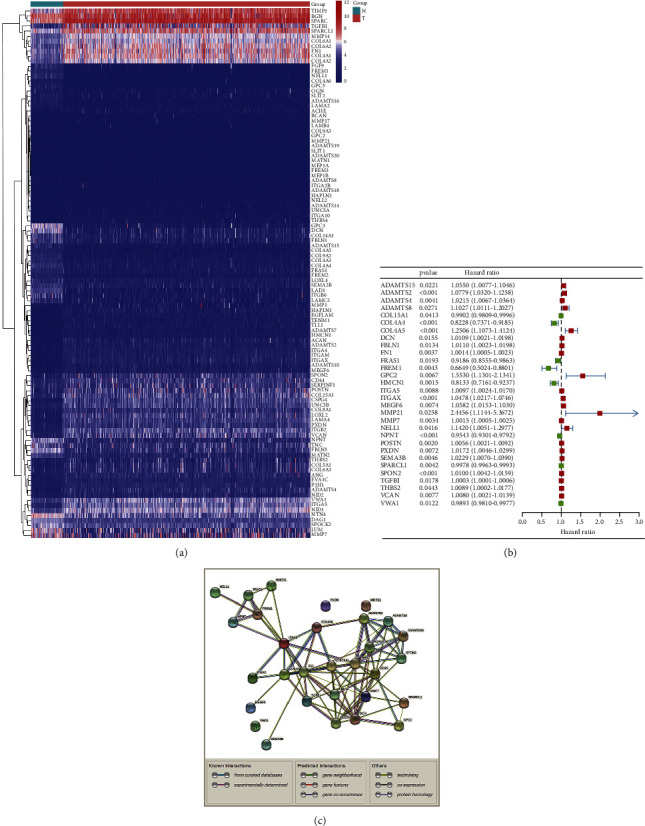
Identification of the candidate BM-related genes in the TCGA cohort. (a) Differentially expressed genes associated with BM are shown using a heatmap. (b) BM-related genes having significant predictive value based on OS are visualized in a forest plot. (c) Candidate gene interactions are mapped out by the PPI network retrieved from the STRING database.

As part of the TCGA-KIRC cohort, 541 people with ccRCC were enrolled. We collected 224 BM-related genes. 106 BM-related genes were identified as DEGs between ccRCC samples and paracancer samples (FDR < 0.05; Figure 1(a)). The univariate Cox regression analysis showed that 30 BM-related DEGs were correlated with OS (Figure 1(b)). Interactions of BM-related genes were visualized with the PPI networks of the differentially expressed BMs comprising 30 nodes and 82 edges (Figure 1(c)).

### 3.2. Construction of a Prognostic Model for BM-Related Risk Score

**Figure 2:**
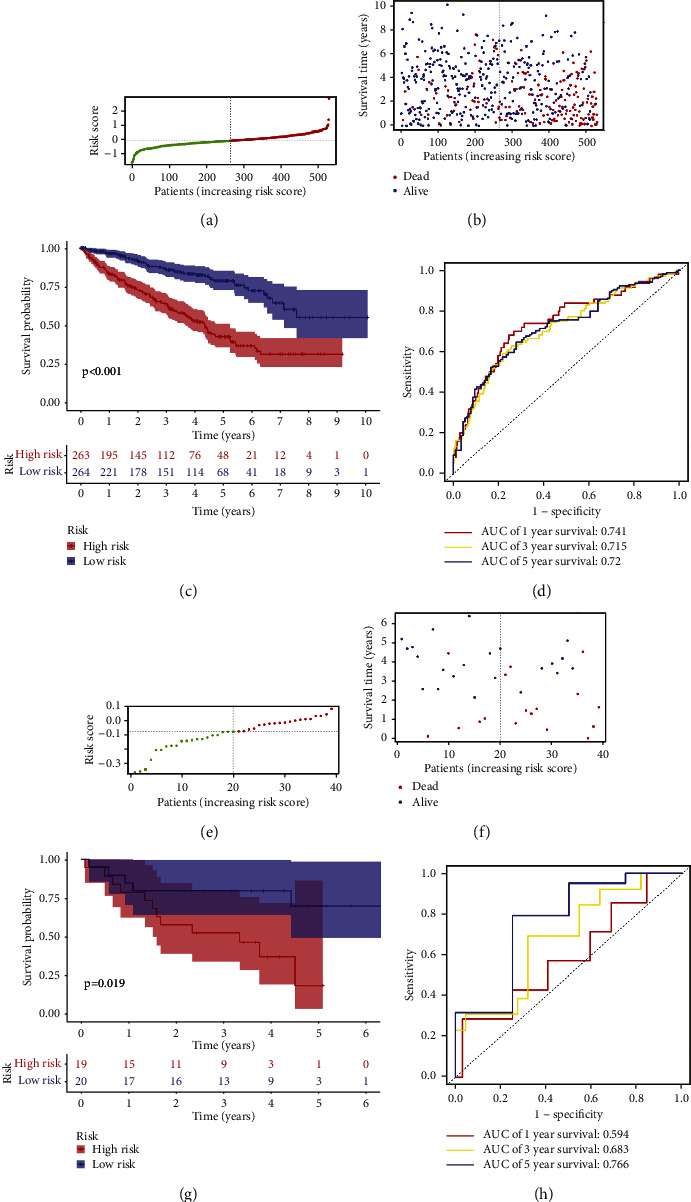
Distribution and prognostic analyses of the 20-gene signature in the TCGA cohort and GSE29609 cohort. (a, b) The distributions of the risk scores and corresponding survival status of KIRC patients in the TCGA cohort. (c) KM curves for the OS of ccRCC patients in the high- and low-risk group in the TCGA cohort. (d) The AUC of time-dependent ROC curves confirmed the risk score's prognostic efficacy in the TCGA cohort. (e, f) The distributions of the risk scores and corresponding survival status of the GSE29609 dataset. (g) KM curves for the OS of patients in the high- and low-risk groups in the GSE29609 dataset. (h) The AUC of time-dependent ROC curves confirmed the risk score's prognostic efficacy in the GSE29609 dataset.

With the expression profiles of the 30 genes mentioned above, we identified a 20-gene prognostic model by LASSO Cox regression analysis (Supplementary Figure S1 A-B). According to the median of the risk score (Risk score = (0.0143)*∗*ADAMTS2 + (0.0070)*∗*ADAMTS4 + (0.0135)*∗*ADAMTS8 + (−0.0027)*∗*COL15A1 + (−0.03)35*∗*COL4A4 + (0.1376)*∗*COL4A6 + (0.0038)*∗*DCN + (0.1922)*∗*GPC2 + (−0.1173)*∗*HMCN1 + (0.0026)*∗*ITGA5 + (0.0101)*∗*ITGAX + (0.0137)*∗*MEGF6 + (0.2590)*∗*MMP21 + (0.0003)*∗*MMP7 + (0.0244)*∗*NELL1 + (−0.0181)*∗*NPNT + (0.0109)*∗*PXDN + (0.0022)*∗*SEMA3B + (0.0013)*∗*VCAN + (−0.0048)*∗*VWA1), patients were stratified into high-risk group (*n* = 263) and low-risk groups (*n* = 264) (Figure 2(a)). As shown in Figures 2(b)–2(c), prognosis and risk score were negatively correlated (*p* < 0.001). The defined 20-gene signature was found to be highly effective at predicting the OS for ccRCC patients, as shown by the AUC (AUC = 0.741, 0.715, and 0.720; at 1, 3, and 5 years, respectively, Figure 2(d)). The BM-related genes signature's predictive significance was further verified in the GSE29609 dataset (Figures 2(e)–2(h)). The survival curve confirmed that patients at high risk had a poor prognosis (*p*=0.019; Figure 2(g)). The AUCs were 0.594, 0.683, and 0.766 at 1, 3, and 5 years, according to the time-dependent ROC curve (Figure 2(g)). Particularly, in the high-risk group, the expression of the 14 risk genes rose, whereas the expression of the six protective genes increased in the low-risk group (Supplementary Figure S2).

### 3.3. Independent Prognostic Value of the 20-Gene Signature and Subgroup Analysis

**Figure 3:**
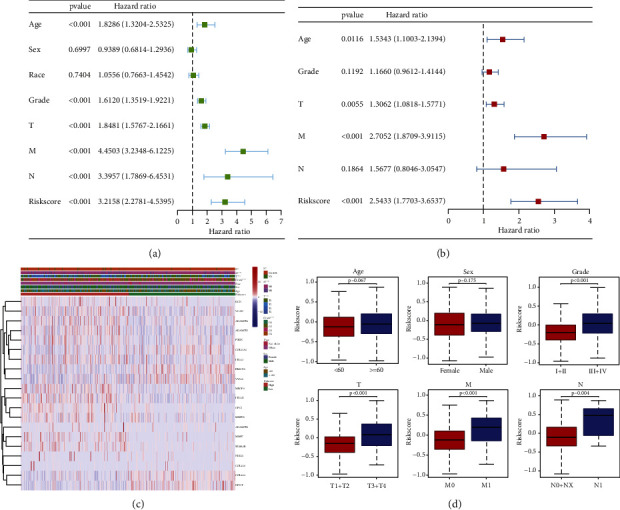
Results of Cox regression for risk factors for ccRCC. (a) Outcomes from a univariate Cox regression study of OS in a cohort of patients with ccRCC based on risk signature score and clinical factors. (b) Results of stepwise multivariate cox regression analysis. (c, d) Correlation of risk group and clinical traits.

**Figure 4:**
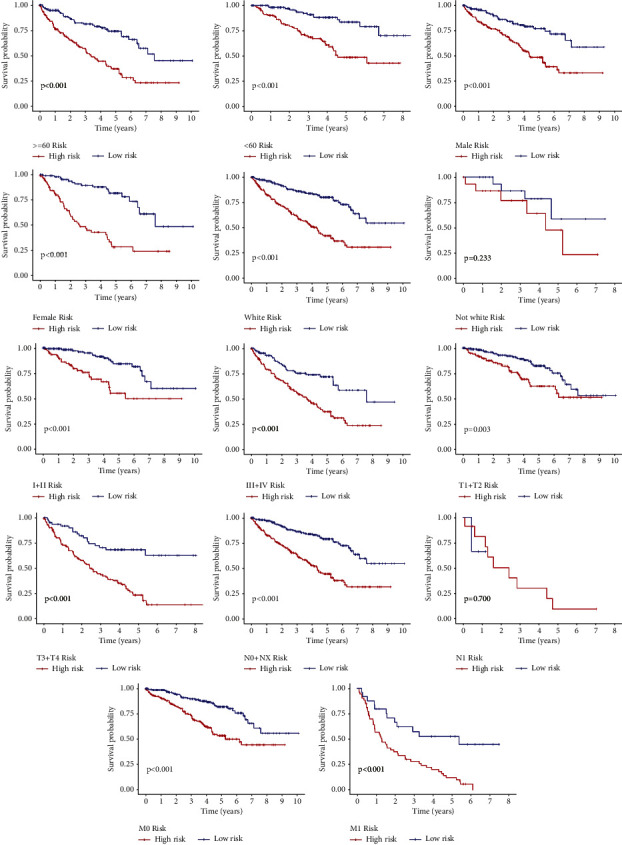
Stratified by age, gender, race, grade, T stage, N stage, or M stage, KM curves demonstrate OS disparities between high- and low-risk groups.

**Figure 5:**
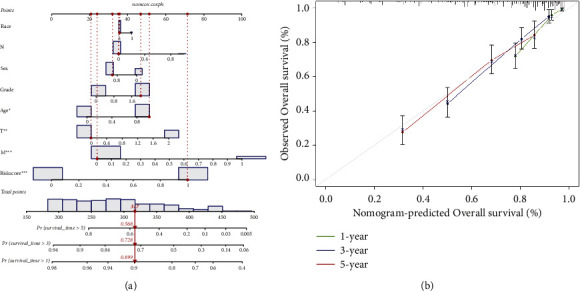
Building a nomogram of 20 BM-related genes. (a) A predictive nomogram for predicting 1, 3, and 5 years OS in ccRCC patients. (b) The calibration plots for predicting 1, 3, 5 years OS.

The independent predictive significance of the 20-gene signature for OS in the risk model was evaluated using multivariate and univariate Cox regression analysis. Univariate cox analysis revealed that risk score, age, grade, and TNM stage are the prognosis-associated factors (*p* < 0.001; Figure 3(a)). In the multivariable competing-risks regression model predicting OS, the risk score is still an independent predictor for OS (Figure 3(b)). Moreover, we stared into whether the prognostic signature was linked to the onset and progression of KIRC. Grade, T stage, N stage, and M stage were all significantly different between high- and low-risk groups (all *p* < 0.001). However, age and gender were not significantly different (*p* > 0.05) (Figures 3(c)–3(d)). Moreover, their prognostic significance in subgroups was also examined by a stratification study. Our research demonstrated that the BM-based signature performed exceptionally well at predicting outcomes in age ≥ 60, age <60, male, female, white, Grade I-II, Grade III-IV, T1-T2, stage T3-T4 stage, N0-NX stage, M0 stage, and M1 stage (all *p* < 0.05). However, BM-related genes have a poor predictive track record in the N1 and not-white populations (*p* > 0.05; Figure 4). All independent predictors of OS in the training cohort were integrated to create the nomogram. The inclusion criteria in the nomogram included risk score, age, gender, race, grade, T stage, N stage, and M stage, as shown in Figure 5(a). The C-indexes for the nomogram predictions were 0.776 (95% CI: 0.742–0.810) for the OS. As indicated by the OS calibration plots, the nomogram might accurately estimate the mortality (Figure 5(b)).

### 3.4. Functional Enrichment Analysis

**Figure 6:**
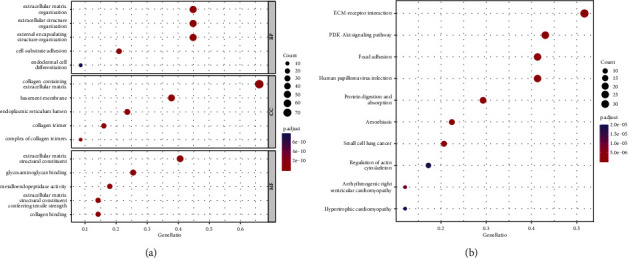
Analyses of GO and KEGG with typical findings. (a) Top 5 significant BP, MF, and CC terms in GO analyses. (b) Top 10 significant KEGG signaling pathways.

20 genes between the high- and low-risk groups were used for GO and KEGG analysis, which shed light on the relationship between risk scores and biological pathways and functions. GO enrichment analysis of the biological process (BP) and molecular functions (MF) showed that DEGs were involved in the tumor cell migration, including cell-substrate adhesion, extracellular matrix structural constituent, and metallopeptidase activity (*p* < 0.05; Figure 6(a)). Additionally, KEGG enrichment analysis revealed that elements related to tumor invasiveness and metastasis, such as ECM-receptor interaction, focal adhesion, and PI3K-Akt signaling pathway, were significantly enriched (*p* < 0.05; Figure 6(b)).

### 3.5. Association between BM-Related Genes and Immune Cells

**Figure 7:**
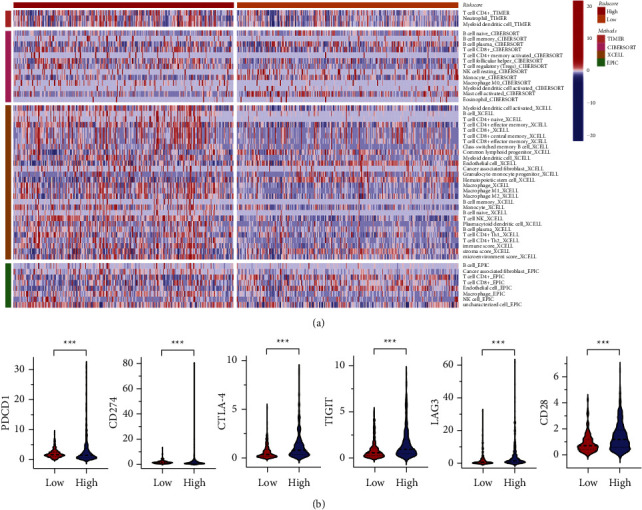
The correlation between BM-related DEGs and immune. (a) Immune cell infiltration between high- and low-risk groups. (b) The connection between prognostic signature and immune checkpoints.

We used TIMER, CIBERSORT, XCELL, and EPIC to investigate the correlation between 20 genes and immune cell infiltration (Figure 7(a)). By CIBERSORT, CD4+ T cells, CD8+ T cells, NK T cells, regulatory T cells (Tregs), B cells, monocytes, macrophages, and dendritic cells had higher immunocyte infiltration degrees in the high-risk group, whereas endothelial cells, mast cells, and hematopoietic stem cells had lower immunocyte infiltration degrees in the low-risk groups. Blocking immune checkpoint pathways is currently thought to be a promising approach to achieving antitumor immunity. We discovered that the expression of PDCD1, CD274, TIGIT, CTLA-4, LAG3, and CD28 was significantly different between the two groups of ccRCC patients (Figure 7(b)). The findings suggest that BM-related genes are actively involved in controlling how the immune system interacts with ccRCC and how their TME develops.

## 4. Discussion

There were 224 BM-related genes examined, and of those, 30 DEGs were shown to be connected with ccRCC prognosis. We used LASSO Cox regression to examine data from the TCGA dataset to identify a 20-gene signature (ADAMTS2, ADAMTS4, ADAMTS8, COL15A1, COL4A4, COL4A6, DCN, GPC2, HMCN1, ITGA5, ITGAX, MEGF6, MMP21, MMP7, NELL1, NPNT, PXDN, SEMA3B, VCAN, and VWA1) in ccRCC patients. In the meanwhile, we used the GSE29609 dataset to validate our risk score and showed that it was effective for predicting ccRCC patients' outcomes. The most important takeaway from our research is the development of a novel BM-based predictive risk profile for ccRCC. This provides a more precise estimation method and a more personalized treatment strategy for the prognosis of ccRCC patients. The risk score is closely related to some clinical features, such as pathological grade and TNM stage. In different subgroups, the vast majority of high-risk groups had worse survival prognoses than lower-risk groups, which reflects the representativeness of BM-related risk scores and has important guiding significance in clinical practice.

In our model, all 20 genes are involved in human cancer occurrence and development, half of which are closely related to RCC (ADAMTS2 [17], COL15A1 [18], COL4A4 [19], DCN [19], ITGA5 [20], ITGAX [21], MMP7 [22], NELL1 [23], SEMA3B [24], and VCAN [25]). The remaining 10 genes still have some papers on their roles in other types of tumors. Cancer development and progression are linked to ADAMTS (a disintegrin and metalloproteinase with thrombospondin motifs) family genes, among which ADAMTS2, 4, and 8 have been shown to have antitumor angiogenesis effects [26–28]. MMP-7 also affects progression by regulating angiogenesis, making it a potential target for RCC [22]. Moreover, the depletion of VCAN also markedly reduced the invasion and migration of cells, which was correlated with MMP7 reduction [25]. It has been reported that deletion of COL15A1 modulates the tumor ECM and leads to increased tumor growth in the mouse mammary carcinoma model [29]. The transcript levels of COL4A4 and 6 could act as potential indicators for early disease progression in ccRCC [30]. Yongcan et al. defined that DCN deficiency promotes RCC growth and metastasis through the downregulation of P21 and E-cadherin [19]. Guoming et al. verified that GPC2, associated with most immune-infiltrating cells, is highly expressed in pan-cancer [31]. ITGA5 and ITGAX are members of the integrin family, commonly used as receptors for the ECM and can be used as a predictor of the prognosis of the RCC in other models. In vitro and in vivo experiments have revealed ccRCC inhibition of SEMA3B associated with methylation through promoter and intronic CpG islands [24]. It is yet unknown how 20-gene signatures play a role in ccRCC.

GO enrichment analysis uncovered that BM-related genes were mainly related to tumor cell migration, such as cell-substrate adhesion, extracellular matrix structural constituent, and metallopeptidase activity. The result of KEGG enrichment analysis indicated that BM-related genes were mostly implicated in focal adhesion, PI3K-Akt signaling pathway, and ECM-receptor interaction. The epithelial-to-mesenchymal transition (EMT), tumor angiogenesis, and changes in the TME are only a few of the multiple mechanisms that contribute to the evolution of mRCC, which is crucially characterized by tumor cell infiltration and metastasis. Cellular signaling pathways, such as PI3K-Akt-mTOR, play a prominent role in pathological conditions of ccRCC. The PI3K-Akt-mTOR pathway could regulate cell proliferation, growth, cell size, metabolism, and motility [32]. EMT is a self-regulated biological process essential for tissue healing in which cells shed their epithelial cell identity and acquire properties of mesenchymal cells. Not only is EMT essential for development and wound healing but it also plays a key role in tumor formation and metastasis.

Although the effects of tumors on the ECM, especially the BM, have been the focus of research over the recent decades, it remains unclear whether tumor immunity is modulated by BM-related genes. We discovered that CD4+ T cells, CD8+ T cells, Tregs, and macrophages were highly enriched in both groups using risk group-based immunological annotation analysis, which may indicate a potential fundamental regulation between tumor immunity and BM. T cells are major players in immune-mediated cancer control and response to immunotherapy. Endothelial BM on the blood and lymphatic vessels is a limiting step for T cell entry into the TME. Besides its well-documented functions in promoting tumor neoangiogenesis, BMs have also been proposed to regulate the function of T cells. BM not only regulates T cell adhesion and migration but also directly regulates T cell activation, function, proliferation, and survival. Evidence suggests that Tregs have antitumor immunity, and an increased density of macrophages is related to poor clinical prognosis in ccRCC. M2-like macrophages can degrade the tumor ECM, destroy the BM, and recruit immunosuppressor cells, all of which further promote tumor progression and distal metastasis. Currently, a variety of innovative immunotherapies based on targeting immune checkpoint inhibitors (ICIs) are in clinical development and are used to treat mRCC patients, which was consistent with our results that the expression of PD-1, PD-L1, CTLA-4, TIGIT, LAG3, and CD28 have a prominent difference between the two groups. Despite multiple lines of evidence elucidating the functions of diverse immune cells and ICIs in cancer, the underlying mechanisms remain poorly characterized in ccRCC and are lacking in the field of BMs.

In the present study, we shed light on the involvement of BMs in ccRCC and developed a promising risk-prognostic signature. In both the derivation and validation cohorts, this model was found to be independently linked with OS. Research in the molecular underpinnings of tumor immunity in ccRCC has been hampered by a lack of knowledge about the relationship between tumor-associated BM genes and the immune system.

## Data Availability

Data are available from the corresponding author upon reasonable request.

## Supplementary Materials

Supplementary Materials Figure S1 Construction of a BM-related genes risk signature. (A) LASSO coefficient profiles of the 30 DEGs. (B) Ten-foldcross-validation was used to select tuning parameters (*λ*) in the lasso model. Figure S2 Heatmap for the differences of 20 BM-related genes between high- and low-risk patients. (A) The TCGA cohort's differences in 20 BM-related genes between high- and low-risk patients. (B) The GSE29609 cohort's differences in 20 BM-related genes between high- and low-risk patients. Table S1. BM-related genes.
