# Supplementary material for: A 20-Gene Signature Predicting Survival in Patients with Clear Cell Renal Cell Carcinoma Based on Basement Membrane
Source: J Oncol. 2023 Apr 8;2023:1302278. doi: 10.1155/2023/1302278 (PMC10118896; doi:10.1155/2023/1302278)
Supplement: Supplementary Materials — Figure S1 Construction of a BM-related genes risk signature. (A) LASSO coefficient profiles of the 30 DEGs. (B) Ten-foldcross-validation was used to select tuning parameters (λ) in the lasso model. Figure S2 Heatmap for the differences of 20 BM-related genes between high- and low-risk patients. (A) The TCGA cohort's differences in 20 BM-related genes between high- and low-risk patients. (B) The GSE29609 cohort's differences in 20 BM-related genes between high- and low-risk patients. Table S1. BM-related genes. [file 1302278.f1.docx]

**Supplementary**


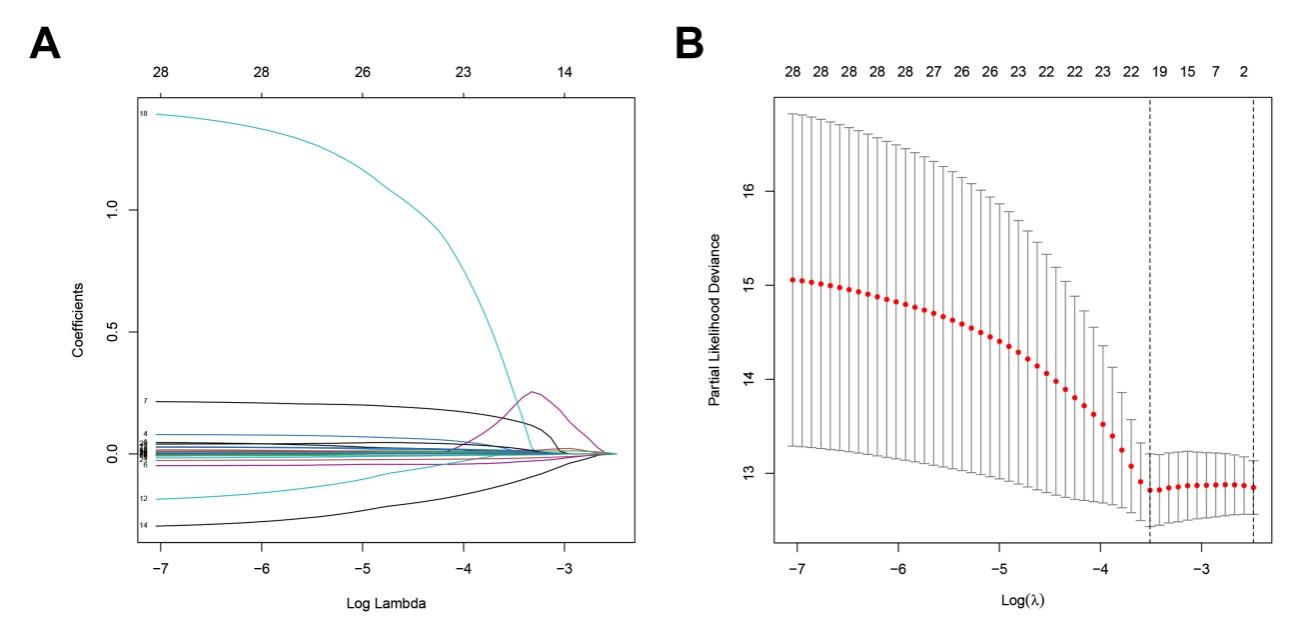


**Figure S1** Construction of a BM-related genes risk signature. (A) LASSO coefficient profiles of the 30 DEGs. (B) Ten-fold cross-validation was used to select tuning parameters (λ) in the lasso model.


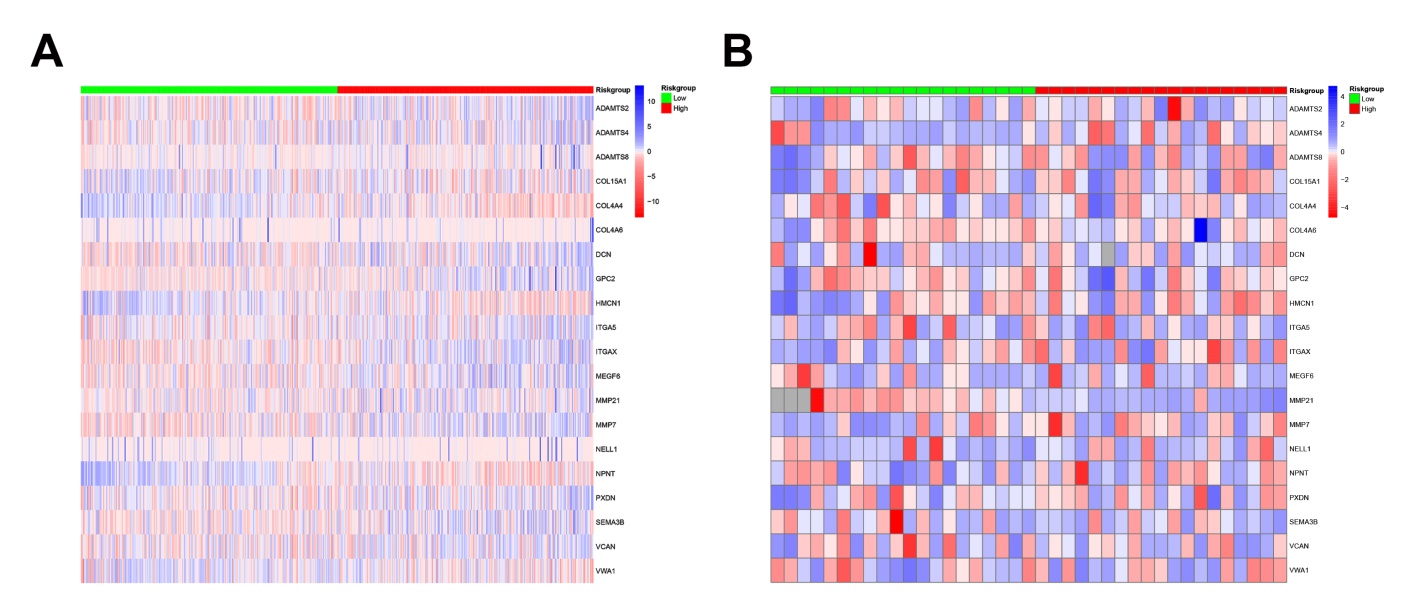


**Figure S2** Heatmap for the differences of 20 BM-related genes between high- and low-risk patients. (A) The TCGA cohort's differences in 20 BM-related genes between high- and low-risk patients. (B) The GSE29609 cohort's differences in 20 BM-related genes between high- and low-risk patients.

Table S1. BM-related genes

| NO. | ID | NO. | ID | NO. | ID | NO. | ID |
| --- | --- | --- | --- | --- | --- | --- | --- |
| 1 | ACAN | 57 | FREM1 | 113 | ACHE | 169 | LOXL4 |
| 2 | ADAM10 | 58 | FREM2 | 114 | ADAMTS1 | 170 | LUM |
| 3 | ADAM17 | 59 | GPC3 | 115 | ADAMTS14 | 171 | MATN1 |
| 4 | ADAM9 | 60 | GPC4 | 116 | ADAMTS15 | 172 | MATN2 |
| 5 | ADAMTS10 | 61 | GPC6 | 117 | ADAMTS16 | 173 | MATN4 |
| 6 | ADAMTS13 | 62 | HMCN1 | 118 | ADAMTS19 | 174 | MEGF6 |
| 7 | ADAMTS17 | 63 | HSPG2 | 119 | ADAMTS20 | 175 | MEGF9 |
| 8 | ADAMTS18 | 64 | ITGA2B | 120 | ADAMTS4 | 176 | MEP1A |
| 9 | ADAMTS2 | 65 | ITGA3 | 121 | ADAMTS5 | 177 | MEP1B |
| 10 | ADAMTS3 | 66 | ITGA6 | 122 | ADAMTS6 | 178 | MMP17 |
| 11 | AGRN | 67 | ITGA7 | 123 | ADAMTS7 | 179 | MMP26 |
| 12 | AMELX | 68 | ITGA8 | 124 | ADAMTS8 | 180 | MMP7 |
| 13 | AMTN | 69 | ITGB2 | 125 | ADAMTS9 | 181 | MMRN2 |
| 14 | ANG | 70 | ITGB3 | 126 | BCAN | 182 | NELL1 |
| 15 | BGN | 71 | ITGB4 | 127 | CCDC80 | 183 | NELL2 |
| 16 | CD151 | 72 | ITGB6 | 128 | CD44 | 184 | NID1 |
| 17 | CERT1 | 73 | LAMA1 | 129 | COL14A1 | 185 | NID2 |
| 18 | COL12A1 | 74 | LAMA2 | 130 | COL15A1 | 186 | NPNT |
| 19 | COL13A1 | 75 | LAMA3 | 131 | COL28A1 | 187 | NTN4 |
| 20 | COL17A1 | 76 | LAMA4 | 132 | COL8A1 | 188 | OGN |
| 21 | COL18A1 | 77 | LAMB1 | 133 | CSPG4 | 189 | OPTC |
| 22 | COL2A1 | 78 | LAMB2 | 134 | DDR1 | 190 | PAPLN |
| 23 | COL4A1 | 79 | LAMB3 | 135 | EGFL6 | 191 | PHF13 |
| 24 | COL4A2 | 80 | LAMC2 | 136 | EGFLAM | 192 | PODN |
| 25 | COL4A3 | 81 | LAMC3 | 137 | EVA1A | 193 | POSTN |
| 26 | COL4A4 | 82 | LOXL1 | 138 | EVA1B | 194 | PTN |
| 27 | COL4A5 | 83 | MMP1 | 139 | EVA1C | 195 | PXDNL |
| 28 | COL4A6 | 84 | MMP14 | 140 | FBLN2 | 196 | RECK |
| 29 | COL5A1 | 85 | MMP2 | 141 | FBN3 | 197 | ROBO1 |
| 30 | COL6A1 | 86 | MMP21 | 142 | FMOD | 198 | SDC1 |
| 31 | COL6A2 | 87 | MPZL2 | 143 | FREM3 | 199 | SDC4 |
| 32 | COL6A3 | 88 | MUSK | 144 | GPC1 | 200 | SEMA3B |
| 33 | COL7A1 | 89 | NTN1 | 145 | GPC2 | 201 | SLIT1 |
| 34 | COL8A2 | 90 | P3H1 | 146 | GPC5 | 202 | SLIT2 |
| 35 | COL9A1 | 91 | P3H2 | 147 | HAPLN1 | 203 | SLIT3 |
| 36 | COL9A2 | 92 | PTPRF | 148 | HAPLN2 | 204 | SPARCL1 |
| 37 | COL9A3 | 93 | PXDN | 149 | HMCN2 | 205 | SPOCK1 |
| 38 | COLQ | 94 | ROBO2 | 150 | ISLR | 206 | SPOCK2 |
| 39 | CST3 | 95 | ROBO3 | 151 | ITGA1 | 207 | SPOCK3 |
| 40 | CTSA | 96 | ROBO4 | 152 | ITGA10 | 208 | SPON1 |
| 41 | CTSB | 97 | RPSA | 153 | ITGA2 | 209 | SPON2 |
| 42 | CTSD | 98 | SERPINF1 | 154 | ITGA4 | 210 | TENM1 |
| 43 | DAG1 | 99 | SMC3 | 155 | ITGA5 | 211 | TENM2 |
| 44 | DCC | 100 | SMOC1 | 156 | ITGA9 | 212 | THBS1 |
| 45 | DCN | 101 | SMOC2 | 157 | ITGAM | 213 | THBS2 |
| 46 | DDR2 | 102 | SPARC | 158 | ITGAV | 214 | THBS4 |
| 47 | ECM1 | 103 | TENM3 | 159 | ITGAX | 215 | TIMP1 |
| 48 | EFEMP1 | 104 | TENM4 | 160 | ITGB1 | 216 | TIMP2 |
| 49 | EFEMP2 | 105 | TGFB1 | 161 | ITGB5 | 217 | TINAG |
| 50 | FBLN1 | 106 | TGFB2 | 162 | ITGB7 | 218 | TINAGL1 |
| 51 | FBLN5 | 107 | TGFBI | 163 | ITGB8 | 219 | UNC5A |
| 52 | FBN1 | 108 | TIMP3 | 164 | LAD1 | 220 | UNC5B |
| 53 | FBN2 | 109 | TLL1 | 165 | LAMA5 | 221 | UNC5C |
| 54 | FGF9 | 110 | TNC | 166 | LAMB4 | 222 | UNC5D |
| 55 | FN1 | 111 | USH2A | 167 | LAMC1 | 223 | VTN |
| 56 | FRAS1 | 112 | VCAN | 168 | LOXL2 | 224 | VWA1 |
